# Cardioprotective effects of vaccination in hospitalized patients with COVID-19

**DOI:** 10.1007/s10238-024-01367-3

**Published:** 2024-05-17

**Authors:** Julian Madrid, Prerana Agarwal, Katharina Müller-Peltzer, Leo Benning, Mischa Selig, Bernd Rolauffs, Philipp Diehl, Johannes Kalbhenn, Georg Trummer, Stefan Utzolino, Tobias Wengenmayer, Hans-Jörg Busch, Daiana Stolz, Siegbert Rieg, Marcus Panning, Fabian Bamberg, Christopher L. Schlett, Esther Askani

**Affiliations:** 1 Department of Cardiology, Pneumology, Angiology, Acute Geriatrics and Intensive Care, Ortenau Klinikum, Klostenstraße 19, 77933 Lahr/Schwarzwald, Germany; 2 Department of Diagnostic and Interventional Radiology, Medical Center – University of Freiburg, Faculty of Medicine, University of Freiburg, Hugstetter Str. 55, 79106 Freiburg, Germany; 3 University Emergency Center, Medical Center – University of Freiburg, Faculty of Medicine, University of Freiburg, Freiburg, Germany; 4 G.E.R.N. Research Center for Tissue Replacement, Regeneration & Neogenesis, Department of Orthopedics and Trauma Surgery, Medical Center – University of Freiburg, Faculty of Medicine, University of Freiburg, Freiburg, Germany; 5 Department of Anesthesiology and Intensive Care Medicine, Medical Center – University of Freiburg, Faculty of Medicine, University of Freiburg, Freiburg, Germany; 6 Department of Cardiovascular Surgery, Medical Center – University of Freiburg, Faculty of Medicine, University of Freiburg, Freiburg, Germany; 7 Department of General and Visceral Surgery, Medical Center – University of Freiburg, Faculty of Medicine, University of Freiburg, Freiburg, Germany; 8 Interdisciplinary Medical Intensive Care, Medical Center – University of Freiburg, Faculty of Medicine, University of Freiburg, Freiburg, Germany; 9 Clinic of Respiratory Medicine, Medical Center – University of Freiburg, Faculty of Medicine, University of Freiburg, Freiburg, Germany; 10 Division of Infectious Diseases, Department of Medicine II, Medical Center –University of Freiburg, Faculty of Medicine, University of Freiburg, Freiburg, Germany; 11 Institute of Virology, Medical Center – University of Freiburg, Faculty of Medicine, University of Freiburg, Freiburg, Germany

**Keywords:** COVID-19 disease, Vaccination, Acute cardiac events, Troponin, Cardiotropism, Biomarker

## Abstract

COVID-19 vaccination has been shown to prevent and reduce the severity of COVID-19 disease. The aim of this study was to explore the cardioprotective effect of COVID-19 vaccination in hospitalized COVID-19 patients. In this retrospective, single-center cohort study, we included hospitalized COVID-19 patients with confirmed vaccination status from July 2021 to February 2022. We assessed outcomes such as acute cardiac events and cardiac biomarker levels through clinical and laboratory data. Our analysis covered 167 patients (69% male, mean age 58 years, 42% being fully vaccinated). After adjustment for confounders, vaccinated hospitalized COVID-19 patients displayed a reduced relative risk for acute cardiac events (RR: 0.33, 95% CI [0.07; 0.75]) and showed diminished troponin T levels (Cohen’s d: − 0.52, 95% CI [− 1.01; − 0.14]), compared to their non-vaccinated peers. Type 2 diabetes (OR: 2.99, 95% CI [1.22; 7.35]) and existing cardiac diseases (OR: 4.31, 95% CI [1.83; 10.74]) were identified as significant risk factors for the emergence of acute cardiac events. Our findings suggest that COVID-19 vaccination may confer both direct and indirect cardioprotective effects in hospitalized COVID-19 patients.

**Supplementary Information:**

The online version contains supplementary material available at 10.1007/s10238-024-01367-3.

## Introduction

The coronavirus disease (COVID-19), caused by SARS-CoV-2, was initially identified at the end of 2019 and quickly escalated into a global pandemic [1]. Despite challenges posed by the virus's evolution, the development of vaccines has played a key role in lessening the pandemic's severity [2].

The detailed pathophysiology of COVID-19 is still being explored. Severe cases are often marked by an uncontrolled immune reaction, leading to a cytokine storm [3]. Moreover, the direct impact of the COVID-19 infection spans multiple organs, which may be a result of virus tropism, suggesting a varied risk profile for different body systems [4].

**Fig. 1:**
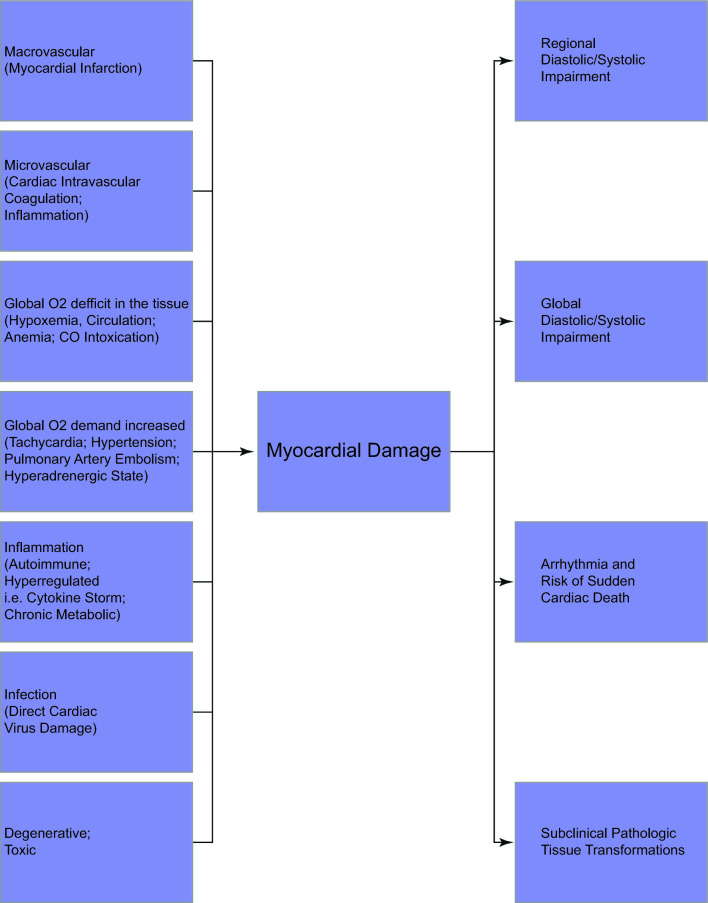
Histopathological and clinical manifestations of myocardial damage [8, 9]

COVID-19 presents not only as a respiratory and systemic condition, but also has distinct effects on various organs, corresponding to specific organ tropisms. These effects are notably evident in the cardiovascular system, where 20–30% of patients exhibit myocardial injury, indicated by elevated troponin levels [5, 6]. Furthermore, COVID-19 infection heightens the risk of acute cardiac events, including myocardial infarction, heart failure, arrhythmia, and myocarditis [5, 6]. The underlying mechanisms of cardiac system damage are likely multifaceted and challenging to distinguish, even with postmortem examinations [5, 7]. It is crucial to discern between indirect harm from systemic reactions following a COVID-19 infection and direct damage due to the virus's cardiotropism. The various contributors to myocardial damage are depicted in Fig. 1 [8, 9], with many of the indirect factors potentially precipitating myocardial damage amidst an excessive immune response to COVID-19.

Moreover, a significant direct viral affinity for cardiac tissue is observed in COVID-19 cases, evidenced by the presence of viral RNA in cardiomyocytes in 35% of patients in postmortem studies [5, 10]. This cardiotropism is likely facilitated by the abundance of ACE2 receptors, notably concentrated in the heart and lungs [11]. Research has demonstrated that COVID-19 vaccination is effective in preventing infection from the virus [12, 13]. As such, vaccination may confer direct or indirect cardioprotective benefits, potentially leading to a reduction in acute cardiac events among hospitalized COVID-19 patients. Yet, as of now, no research has directly compared the incidence of acute cardiac events and cardiac biomarker levels between vaccinated and unvaccinated hospitalized patients infected with COVID-19. Given that cardiac damage significantly affects patients' long-term health [14], it is crucial to assess the impact of COVID-19 vaccination on cardiac health.

Our research examined the impact of COVID-19 vaccination on the frequency of acute cardiac events (defined as myocardial infarction, heart failure, arrhythmia, and myocarditis) as the primary outcome and the amount of cardiac damage assessed through levels of cardiac biomarkers as secondary outcomes, among hospitalized COVID-19 patients.

## Methods

**Fig. 2:**
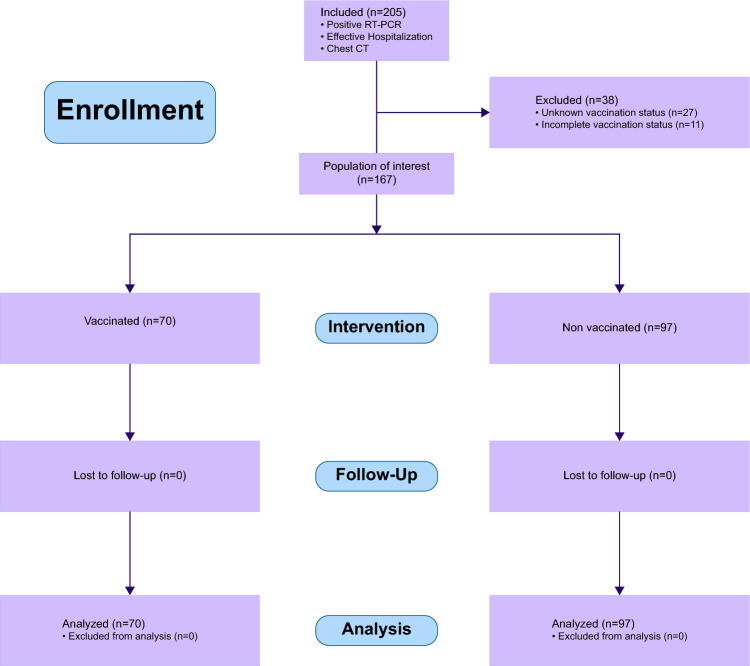
Flow diagram: enrollment of study participants

In this retrospective cohort analysis, we evaluated a patient group admitted for COVID-19 infection. We focused on their vaccination status and looked at the incidence of acute cardiac events as the primary outcome, with the vaccination's influence on cardiac biomarkers as secondary outcomes. From July 2021 onward, the hospital documented the vaccination status of all in-patients. This study included individuals hospitalized for COVID-19 from July 1, 2021, to February 14, 2022, confirmed by a positive RT-PCR test and at least one chest computed tomography (CT) scan during their stay. Exclusions were made for patients with incomplete vaccination data, those only partially vaccinated, or those younger than 18 years old, as shown in Fig. 2.

### Ethical statements

This investigation was granted ethical clearance by the Institutional Research Ethics Board of the Medical Faculty at the Albert-Ludwig-University Freiburg. Due to its retrospective nature, the Ethics Committee of the Medical Faculty at the same university waived the requirement for informed consent. All procedures were aligned with the Helsinki Declaration's guidelines for research involving human subjects.

### Vaccination classification

Vaccination status in this study was delineated into three distinct groups: those not vaccinated, those partially vaccinated, and those fully vaccinated. Unvaccinated individuals were identified as those without any documented COVID-19 vaccination. Partial vaccination was attributed to individuals who had received only the first vaccine dose or contracted COVID-19 less than 2 weeks after their second dose. Conversely, individuals were considered fully vaccinated if they contracted COVID-19 or manifested symptoms at least 2 weeks following the administration of their second vaccine dose. If vaccination records were missing or if the date of the last vaccine was unclear, the vaccination status was classified as unknown. The study did not distinguish between different vaccine types (such as AstraZeneca's ChAdOx1 nCoV-19, Pfizer-BioNTech's BNT162b2, or Moderna's mRNA-1273), except in one scenario. Individuals initially vaccinated with Johnson & Johnson-Janssen's Ad26.COV2. They were considered fully vaccinated only if they had received a subsequent mRNA vaccine dose at least 14 days before testing positive for COVID-19 or displaying symptoms.

### Data acquisition

Upon establishing the study group using the hospital's electronic information system, relevant clinical information was collected from the patient's digital health records. The collected data encompassed details on vaccination status, demographic information, characteristics of the infection such as the type of virus and the onset of symptoms, clinical signs, existing health conditions including body mass index (BMI) and prior diseases, laboratory test results, electrocardiogram, echocardiography, CT scan findings, MRI findings, coronary angiography, specifics of the treatment for COVID-19, and any ensuing complications.

Acute cardiac events were defined as follows: myocardial infarction, heart failure, arrhythmia, and myocarditis. The amount of cardiac damage was assessed through levels of cardiac biomarkers (Troponin T, N-terminal pro b-type natriuretic peptide (nt pro-BNP), Creatine kinase (CK), CK-MB, Lactate dehydrogenase (LDH)).

The general extent of COVID-19 lung parenchymal involvement was semi-quantitatively scored for each pulmonary lobe: visual involvement of less than 1/3rd of lobar volume (score 1), visual involvement of 1/3rd to 2/3rd of lobar volume (score 2), visual involvement of more than 2/3rd of lobar volume (score 3); the scores of the individual lobes were then added together (maximum possible score for both lungs being 15) [15, 16]. Chest CT scans were also analyzed for the presence or absence of coronary calcification, pericardial effusion, and in case of contrast-enhanced CT-scans pulmonary embolism.

### Statistical analysis

We analyzed how patients' initial conditions varied based on whether they were vaccinated or not, using logistic regression. We adjusted for major differences in demographics between the groups to ensure they did not skew the main results. However, some rare demographic factors, although not common in the larger group, showed differences between vaccinated and non-vaccinated groups. Factors that rarely occur are not reliable for use as confounding variables in the analysis due to the potential for causing numerical instability in the regression results. For this reason, we carried out an additional sub-group analysis for all significant demographic factors that occurred rarely, less than 10 times [17]. We then performed the same analysis on the group that did not include these rare factors. Hence, we analyzed the sensitivity of the results stratified by the presence of rare characteristics. Consequently, it remains possible to make a generalized statement about the broader population without the influence of these infrequent characteristics.

In assessing the primary outcome, we conducted logistic regression analyses, both unadjusted and adjusted for potential confounders, including age, type 2 diabetes, hypertension, cardiac, and pulmonary diseases. We performed similar analyses within subgroups characterized by infrequent occurrences, such as different virus variants, preexisting conditions, immunodeficiency, cancer, vascular and neurological diseases, organ transplants, and pregnancy. Due to the scarcity of these events, we cannot draw definitive conclusions about the vaccine's effectiveness for these specific conditions.

To ascertain the association between vaccination status and indicators of cardiac damage, such as cardiac biomarkers, pericardial effusion, and pulmonary embolism, we utilized both logistic and linear regression models, accounting for potential confounders including age, type 2 diabetes, hypertension, cardiac, and pulmonary diseases. In our analysis of laboratory parameters, we adjusted for the duration since symptom onset. Given that certain biomarkers, notably troponin T, are renally excreted and consequently closely linked with renal function, we made adjustments for creatinine levels and excluded participants presenting with exceptionally elevated creatinine levels.

In our research, we conducted a stratified analysis of the primary outcome, differentiating between two age cohorts: those under 60 years and those over 60 years of age. We specifically evaluated the vaccine's impact on acute cardiac events and troponin T levels, applying adjustments for the aforementioned confounding factors.

To identify significant predictors of acute cardiac events in patients admitted with COVID-19, we implemented a regularized logistic regression using the least absolute shrinkage and selection operator (LASSO) technique [18]. We then conducted logistic regression analyses for each predictor identified, adjusting for the patient's vaccination status.

We utilized both logistic and linear regression methods to investigate the association between acute cardiac events and various clinical characteristics, while adjusting for potential vaccination status. Additionally, in our analysis of laboratory data, we accounted for the duration since initial symptom onset.

Furthermore, we investigated the relationship between several other parameters: acute cardiac events versus troponin T levels; ARDS versus acute cardiac events; ARDS versus troponin T levels; mortality versus acute cardiac events; mortality versus troponin T levels; and the effect of coronary calcification on acute cardiac events and troponin T levels.

We calculated all effect sizes and adjusted effect sizes with 95% confidence intervals (CI) using a bootstrapping technique in tandem with the generalized linear model framework. In the context of logistic regression, we determined the relative risk (RR) ratios and their adjusted equivalents when possible; alternatively, we estimated odds ratios (OR) and their adjusted versions. Within the linear regression analysis, we measured Cohen's d along with its adjusted form as relevant measures of effect size [19].

We assessed statistical significance through confidence intervals: RR ratios and ORs were considered significant if their 95% CI did not include 1, while Cohen's d was significant if its 95% CI did not include 0. To ensure the validity of our findings, we examined residual plots and q-q plots, checking for the independence of observations, absence of residual trends, linearity, normal distribution of data, homoscedasticity, and the presence of outliers. Descriptive statistics were systematically presented: mean values and their 95% CIs for continuous variables were estimated via bootstrapping methods; for categorical variables, we reported frequencies and percentages. We conducted a post hoc power sensitivity analysis and determined the smallest relative risk (RR) ratio detectable in a study with adequate power (> 80%) for the primary outcome. Given a sample size of 167 participants, the study was sufficiently powered (> 80%) to detect a RR smaller than 0.78 and greater than 1.22. All statistical analyses were performed using RStudio (v 2023.06.0 + 421, RStudio, Inc.).

## Results

A cohort of 167 patients, who were admitted for COVID-19 infection between July 1, 2021, and February 14, 2022, to a tertiary referral hospital, was analyzed. They were mainly between 55 and 60 years old and males were twice as frequent as female patients. Delta was the dominant virus variant (78%) followed by Omicron (22%) in patients undergoing virus testing (*n* = 81). Within the entire cohort 42% (*n* = 70) were vaccinated. Further demographics are summarized in Table 1.

**Table 1:** Demographic characteristics according to COVID-19 vaccination status

	All	Vaccination Status (*n* = 167)		Odds Ratio (± 95% CI)
		Non-vaccinated (*n* = 97)	Vaccinated (*n* = 70)	
General information				
Age (years) (*n* = 167)	57.86	51.10	67.21	*1.074 (1.048;1.102)*
	(55.33; 60.36)	(48.12; 54.04)	(63.97; 70.59)	
Sex *n* = (167)				1.09 (0.56; 2.15)
Male	115 (69%)	66 (68%)	49 (70%)	
Female	52 (31%)	31 (32%)	21 (30%)	
Virus variant (*n* = 81)**				*7.11 (2.09; 33.00)*
Omicron	18 (22%)	3 (8%)	15 (37%)	
Delta	63 (78%)	37 (92%)	26 (63%)	
Pre-existing conditions				
BMI (*n* = 129)				0.75 (0.36; 1.57)
> 25 kg/m^2^	86 (67%)	50 (69%)	36 (42%)	
< 25 kg/m^2^	43 (33%)	22 (31%)	50 (58%)	
Pregnancy (*n* = 167)*				*0.0 (0.0; 0.0)*
Yes	6 (3.5%)	6 (6%)	0 (0%)	
No	161 (96.5%)	91 (94%)	70 (100%)	
Pre-existing diseases (*n* = 167)**				*7.75 (2.87; 27.14)*
Yes	132 (79%)	66 (68%)	66 (94%)	
No	35 (21%)	31 (32%)	4 (6%)	
Immunodeficiency (through disease or medication) (*n* = 167)**				*4.78 (1.96;12.97)*
Yes	26 (16%)	7 (7%)	19 (27%)	
No	141 (84%)	90 (93%)	51 (73%)	
Prediabetic metabolism (*n* = 167)*				0.45 (0.02; 3.63)
Yes	4 (2%)	3 (3%)	1 (1%)	
No	163 (98%)	94 (97%)	69 (99%)	
Type 2 diabetes (*n* = 167)				*2.83 (1.29; 6.43)*
Yes	32 (19%)	12 (12%)	20 (29%)	
No	135 (81%)	85 (88%)	50 (71%)	
Hypertension (*n* = 167)				*4.07 (2.12; 7.96)*
Yes	66 (40%)	25 (26%)	41 (59%)	
No	101 (60%)	72 (74%)	29 (41%)	
Anemia (*n* = 167)				1.32 (0.53; 3.21)
Yes	23 (14%)	12 (12%)	11 (16%)	
No	144 (86%)	85 (88%)	59 (84%)	
Rheumatological disease (*n* = 167)*				1.41 (0.37; 5.28)
Yes	10 (6%)	5 (5%)	5 (7%)	
No	157 (94%)	92 (95%)	65 (93%)	
Oncological disease (*n* = 167)**				*6.50 (2.59; 18.68)*
Yes	27 (16%)	6 (6%)	21 (30%)	
No	140 (84%)	91 (94%)	49 (70%)	
Infectious disease (*n* = 167)*				0.37 (0.05; 1.62)
Yes	9 (5%)	7 (7%)	2 (3%)	
No	158 (95%)	90 (93%)	68 (97%)	
Cardiac disease (*n* = 167)				*4.98 (2.50; 10.26)*
Yes	53 (32%)	17 (18%)	36 (51%)	
No	114 (68%)	80 (82%)	34 (49%)	
Vascular disease (*n* = 167)**				*3.56 (1.48; 9.27)*
Yes	25 (15%)	8 (8%)	17 (24%)	
No	142 (85%)	89 (92%)	53 (76%)	
Pulmonary disease (*n* = 167)				*3.23 (1.56; 6.85)*
Yes	41 (25%)	15 (15%)	26 (37%)	
No	126 (75%)	82 (85%)	44 (63%)	
Neurological disease (*n* = 167)**				*4.13 (1.58; 12.18)*
Yes	21 (13%)	6 (6%)	15 (21%)	
No	146 (87%)	91 (94%)	57 (79%)	
Liver disease (*n* = 167)*				0.57 (0.12; 2.15)
Yes	10 (6%)	7 (7%)	3 (5%)	
No	157 (94%)	90 (93%)	62 (95%)	
Renal disease (*n* = 167)				0.57 (0.12; 2.15)
Yes	38 (23%)	12 (12%)	26 (37%)	
No	129 (77%)	85 (88%)	44 (63%)	
Thyroid disease (*n* = 167)				1.11 (0.51; 2.38)
Yes	34 (20%)	19 (20%)	15 (21%)	
No	133 (80%)	78 (80%)	55 (79%)	
Organ transplant (*n* = 167)**				
Yes	15 (9%)	2 (2%)	13 (19%)	*10.83 (2.85; 70.90)*
No	152 (91%)	95 (98%)	57 (81%)	

Results highlighted in italics are statistically significant

*Rare characteristic but no significant difference between vaccination status and rare characteristic. **Rare characteristic and significant difference between vaccination status and rare characteristic. Body mass index (BMI)

### Demographical differences between vaccinated and unvaccinated patients

Vaccinated patients were on average 16 years older (67 vs. 51 years), and the virus variant Delta was the more dominant (78%). Further, vaccinated patients had a higher prevalence of preexisting diseases. Main differences were with respect to organ transplants (OR: 10.83, 95% CI [2.85; 70.90]), oncological conditions (OR: 6.50, 95% CI [2.59; 18.68]), immunodeficiency status (OR: 4.78, 95% CI [1.96; 12.97]), and cardiac diseases (OR: 4.98, 95% CI [2.50; 10.26]). In the unvaccinated group, there were six instances of pregnancy, while the vaccinated group had none (Table 1).

### Evaluation of vaccination’s protective effect on cardiac damage

**Fig. 3:**
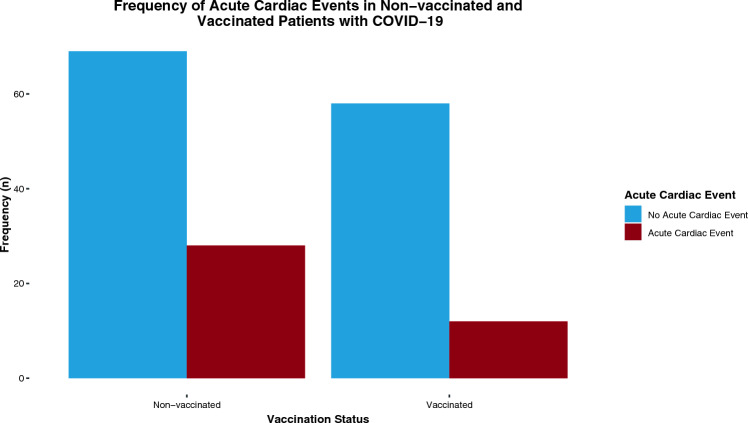
Frequency of acute cardiac events in non-vaccinated and vaccinated patients with COVID-19

After controlling for confounding variables such as age, type 2 diabetes, hypertension, cardiac, and pulmonary diseases, vaccinated individuals experienced significantly reduced acute cardiac events compared to their non-vaccinated counterparts (RR: 0.33, 95% CI [0.07; 0.75]) (Table 2, Fig. 3).

**Table 2:** Primary outcome. Acute cardiac events in vaccinated and non-vaccinated patients with COVID-19

	All (*n* = 167)	Vaccination status		*P*-value	Estimate (± 95% CI)	Risk Ratio (± 95% CI)
		Non-vaccinated	Vaccinated			
Acute Cardiac Events (*n* = 167)				Without correction for confounder variables		
Yes	40 (24%)	28 (29%)	12 (17%)	0.08	− 0.67 (− 1.46; 0.06)	0.61 (0.28; 1.02)
No	127 (76%)	69 (71%)	58 (83%)	With correction for confounder variables		
				*0.0091*	*− 1.36 (− 2.44; − 0.38)*	*0.33 (0.07; 0.75)*

Results highlighted in italics are statistically significant

In subgroup analyses, which did not include patients affected by the omicron variant (RR: 0.26, 95% CI [0.05; 0.64]), as well as those who were healthy (RR: 0.22, 95% CI [0.03; 0.54]), or had no immunodeficiency (RR: 0.46, 95% CI [0.09; 0.99]), no oncological (RR: 0.18, 95% CI [0.01; 0.59]), no vascular (RR: 0.33, 95% CI [0.05; 0.80]), no neurological diseases (RR: 0.37, 95% CI [0.06; 0.91]), had not undergone organ transplants (RR: 0.45, 95% CI [0.09; 0.96]), or were not pregnant (RR: 0.33, 95% CI [0.08; 0.73]), the results remained statistically significant (see Supplementary Table 1, Supplementary Fig. 1).

After accounting for confounders including age, type 2 diabetes, hypertension, cardiac and pulmonary diseases, the duration since symptom onset, and creatinine levels, a significant reduction in troponin T (Cohen’s d: − 0.52, 95% CI [− 1.01; − 0.14]), and LDH levels (Cohen’s d: − 0.46, 95% CI [− 0.81; − 0.11]) were observed among vaccinated subjects in hospitalized COVID-19 patients. Conversely, no statistical differences were noted in the levels of nt-pro-BNP (Cohen’s d: − 0.44, 95% CI [− 0.91; 0.17]), CK (Cohen’s d: − 0.30, 95% CI [− 0.63; 0.14]), and CK-MB (Cohen’s d: − 0.21, 95% CI [− 0.78; 0.68]) when comparing vaccinated to non-vaccinated individuals. Additionally, adjustments for age, type 2 diabetes, hypertension, cardiac, and pulmonary diseases did not reveal any notable effect of vaccination on the occurrence of pericardial effusion (RR: 1.27, 95% CI [0.00; 6000]) or pulmonary embolism (RR: 1.10, 95% CI [0.42; 2.33]) in hospitalized COVID-19 patients (Table 3).

**Table 3:** Secondary outcome. Cardiac characteristics of vaccinated and non-vaccinated patients with COVID-19

		Vaccination status		Without correction	With correction
		Non-vaccinated	Vaccinated	Effect size (± 95% CI)	Aadj Effect size (± 95% CI)
Troponin T (*n* = 99 ng/l)	40.32 (28.8; 58.8)	46.19 (24.8; 71.9)	31.28 (23.6; 40.1)	− 0.19 (− 0.44; 0.19)	*− 0.52 (− 1.01; − 0.14)*
nt pro-BNP (*n* = 104 pg/ml)	946 (612; 1322)	821 (409; 1303)	1182 (673; 1853)	0.18 (− 0.18; 0.66)	− 0.44 (− 0.91; 0.17)
CK (*n* = 129 U/l)	570 (338; 904)	675 (348; 1116)	374 (197; 606)	− 0.18 (− 0.40; 0.12)	− 0.30 (− 0.63; 0.14)
CK-MB (*n* = 43 U/l)	46 (30; 68)	53 (31; 85)	32 (22; 47)	− 0.32 (− 0.68; 0.24)	− 0.21 (− 0.78; 0.68)
LDH (*n* = 119 U/l)	496 (447; 554)	556 (482; 633)	390 (347; 439)	*− 0.57 (− 0.83; − 0.32)*	*− 0.46 (− 0.81; − 0.11)*
Pericardial effusion (*n* = 105)				1.16 (0.17; 5000)	1.27 (0.00; 6000)
Yes	7(37%)	3 (44%)	4 (26%)		
No	98 (63%)	46 (56%)	52 (74%)		
Pulmonary embolism (*n* = 160)				0.97 (0.48; 1.88)	1.10 (0.42; 2.33)
Yes	32 (29%)	19 (35%)	13 (22%)		
No	128 (71%)	75 (65%)	53 (78%)		

Results highlighted in italics are statistically significant

*LDH* Lactate Dehydrogenase,* CK* creatine kinase, creatine kinase (CK)-MB;* nt-pro-BNP* N-terminal pro b-type natriuretic peptide

### Evaluation of vaccination efficacy in age-stratified subgroups

**Fig. 4:**
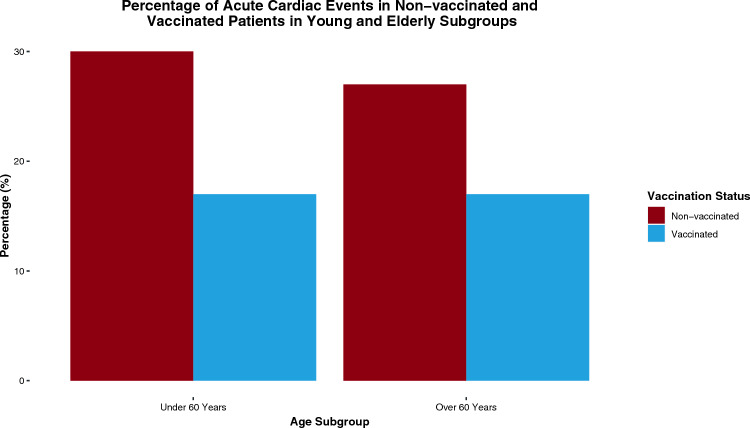
Percentage of acute cardiac events in non-vaccinated and vaccinated patients in young and elderly subgroups

In the subgroup analysis stratified by age, vaccinated individuals under 60 years demonstrated a significant reduction in acute cardiac events compared to their non-vaccinated counterparts when adjusted for confounders such as age, type 2 diabetes, hypertension, and cardiac and pulmonary diseases (RR: 0.16, 95% CI [0.00; 0.77]). However, among those over 60 years, the reduction in acute cardiac events was not statistically significant post-adjustment for these factors (RR: 0.38, 95% CI [0.02; 1.62]) (Fig. 4).

In contrast, within the younger subgroup (aged < 60 years), there was no significant difference in troponin T levels between vaccinated and non-vaccinated patients after adjusting for age, type 2 diabetes, hypertension, cardiac and pulmonary conditions, the duration since the onset of symptoms, and creatinine levels (Cohen’s d: − 0.65, 95% CI [− 1.47; 0.51]). Notably, in the older subgroup (aged > 60 years), a significant decrease in troponin T levels was observed among vaccinated patients as compared to non-vaccinated patients, following adjustments for the same confounders (Cohen’s d: − 0.81, 95% CI [− 1.68; − 0.13]) (Table 4).

**Table 4:** Secondary outcome. Acute cardiac events and Troponin T levels in vaccinated and non-vaccinated patients and young and elderly subgroups

Acute cardiac events in vaccinated and non-vaccinated patients and young and elderly subgroups				
	All (*n* = 167)	Vaccination status		Risk ratio (± 95% CI)
		Non-vaccinated	Vaccinated	
Acute cardiac events in patients < 60 years (*n* = 95)				
Yes	25 (26%)	21 (30%)	4 (17%)	0.56 (0.12; 1.31)
No	70 (74%)	50 (70%)	20 (83%)	
Acute cardiac events in patients > 60 years (*n* = 72)				
Yes	15 (21%)	7 (27%)	8 (17%)	0.64 (0.23; 1.95)
No	57 (79%)	19 (73%)	38 (83%)	

Troponin T levels in vaccinated and non-vaccinated patients and young and elderly subgroups					
	All (*n* = 99)	Vaccination status		Cohen's d (± 95% CI)	Adj. Cohen's d (± 95% CI)
		Non-vaccinated	Vaccinated		
Troponin T in patients < 60 years (*n* = 51)	26.6 (11.9; 50.6)	28.8 (11.0; 58.2)	16.4 (9.7; 24.3)	− 0.16 (− 0.36; 0.62)	− 0.65 (− 1.47; 0.51)
Troponin T in patients > 60 years (*n* = 48)	54.8 (35.8; 79.4)	86.6 (37.9; 136.0)	35.7 (26.2; 47.3)	*− 0.72 (− 1.45; − 0.04)*	− 0.81 (− 1.68; − 0.13)

Results highlighted in italics are statistically significant

### Acute cardiac events and predictive risk factors

To ascertain risk factors associated with the onset of acute cardiac events in COVID-19 patients, we conducted a regularized logistic regression analysis using the LASSO method to account for multicollinearity among variables. This approach is favored over standard logistic regression, as it prevents numerical instability by considering the most relevant correlations between variables. The regularized model was instrumental in identifying the most pertinent independent variables from a broader set, revealing a significant link between acute cardiac events and characteristics such as cardiac disease, type 2 diabetes, immunodeficiency, rheumatological and liver diseases, along vaccination status.

Subsequent logistic regression analyses were performed to estimate the risk associated with each significant characteristic variable, adjusting for vaccination status. Cardiac disease (OR: 4.31, 95% CI [1.83; 10.74]) and type 2 diabetes (OR: 2.99, 95% CI [1.22; 7.35]) emerged as risk factors, while rheumatological disease (OR: 0.0, 95% CI [0.0; 0.0]) appeared to be a protective factor against the development of acute cardiac events. However, age (OR: 1.08, 95% CI [0.72; 1.65]), immunodeficiency (OR: 0.44, 95% CI [0.09; 1.43]), and liver disease (OR: 0.30, 95% CI [0.01; 1.72]) did not achieve statistical significance in this context (Table 5).

**Table 5:** Secondary outcome. Predictive risk factors for acute cardiac events in hospitalized COVID-19 patients

Predictive risk factors for acute cardiac events in COVID-19 patients					
		Acute cardiac events		Without correction for vaccination status	With correction for vaccination status
		No event	Event	Odds ratio (± 95% CI)	Adj Odds ratio (± 95% CI)
Age (years *n* = 167)	57.8 (55.4; 60.2)	58.2 (54.4; 60.8)	56.7 (51.6; 62.1)	0.99 (0.97; 1.01)	1.08 (0.72; 1.65)
Cardiac disease (*n* = 167)				*2.47 (1.18; 5.18)*	*4.31 (1.83; 10.74)*
Yes	53 (32%)	34 (27%)	19 (48%)		
No	114 (68%)	93 (73%)	21 (52%)		
Type 2 diabetes (*n* = 167)				*2.29 (1.03; 5.21)*	*2.99 (1.22; 7.35)*
Yes	32 (19%)	20 (16%)	12 (30%)		
No	135 (81%)	107 (84%)	28 (70%)		
Immunodeficiency (through disease or medication) (*n* = 167)				0.36 (0.08; 1.13)	0.44 (0.09; 1.43)
Yes	26 (16%)	23 (18%)	3 (8%)		
No	141 (84%)	104 (82%)	37 (92%)		
Rheumatological disease (*n* = 167)				*0 (0; 0)*	*0 (0; 0)*
Yes	10 (6%)	10 (8%)	0 (0%)		
No	157 (94%)	117 (92%)	40 (100%)		
Liver disease (*n* = 167)				0.33 (0.01; 1.87)	0.30 (0.01; 1.72)
Yes	10 (6%)	9 (7%)	1 (3%))		
No	157 (94%)	118 (93%)	39 (97%)		

Results highlighted in italics are statistically significant

### Acute cardiac events and associated clinical features

In our study, we further analyzed clinical characteristics linked with COVID-19-related acute cardiac events and adjusted these associations for vaccination status (Table 6). We observed that COVID-19 acute cardiac events were significantly correlated with an increase in non-invasive ventilation (NIV) (OR: 4.97, 95% CI [2.24; 11.53]), the need for intubation and invasive ventilation (OR: 3.55, 95% CI [1.61; 8.01]), extracorporeal membrane oxygenation (ECMO) or extracorporeal lung support (ECLS) (OR: 5.10, 95% CI [2.00; 13.41]), quantitative chest CT lesions (OR: 1.91, 95% CI [1.29; 2.91]), intensive care unit (ICU) therapy (OR: 5.29, 95% CI [2.36; 12.83]), ICU duration (OR: 1.34, 95% CI [1.04; 1.91]), higher mortality (OR: 4.22, 95% CI [1.91; 9.42]), higher sepsis incidence (OR: 4.75, 95% CI [1.98; 11.5]), elevated C-reactive protein (CRP) levels (OR: 1.96, 95% CI [1.33; 3.00]), pulmonary superinfections (OR: 3.10, 95% CI [1.43; 7.11]), increased partial thromboplastin time (PTT) (OR: 1.55, 95% CI [1.09; 2.41]), increased international normalized ratio (INR) (OR: 2.25, 95% CI [1.21; 5.86]), increased platelet counts (OR: 1.46, 95% CI [1.03; 2.10]), increased renal failure occurrence (OR: 3.27, 95% CI [1.57; 6.98]) (Table 6).

**Table 6:** Secondary outcome. Clinical characteristics of COVID-19 patients developing acute cardiac events

		Acute cardiac events		Without correction for vaccination status odds ratio (± 95% CI)	With correction for vaccination status adj odds ratio (± 95% CI)
		No event	Event		
Dyspnea (*n* = 165)				2.93 (0.95; 12.83)	2.56 (0.81; 11.3)
Yes	138 (84%)	101 (81%)	37 (92%)		
No	27 (16%)	24 (19%)	3 (8%)		
Coughing (*n* = 150)				1.71 (0.76; 4.03)	1.80 (0.79; 4.32)
Yes	90 (60%)	67 (31%)	23 (70%)		
No	60 (40%)	50 (69%)	10 (30%)		
Fever (*n* = 149)				1.42 (0.63; 3.40)	1.52 (0.66; 3.68)
Yes	93 (62%)	71 (61%)	22 (69%)		
No	56 (38%)	46 (39%)	10 (31%)		
Oxygen (l/min *n* = 110)	4.20 (3.42; 5.03)	3.90 (3.04; 4.81)	5.55 (3.59; 7.65)	1.07 (0.97; 1.19)	1.39 (0.88; 2.17)
High flow (*n* = 147)				1.73 (0.74; 3.95)	1.65 (0.70; 3.81)
Yes	43 (29%)	31 (27%)	12 (39%)		
No	104 (71%)	85 (73%)	19 (61%)		
NIV (*n* = 153)				*5.31 (2.42; 12.2)*	*4.97 (2.24; 11.53)*
Yes	56 (37%)	32 (27%)	24 (67%)		
No	97 (63%)	85 (73%)	12 (33%)		
Intubation (*n* = 165)				*3.80 (1.80; 8.15)*	*3.55 (1.61; 8.01)*
Yes	54 (33%)	32 (25%)	22 (56%)		
No	111 (67%)	94 (75%)	17 (44%)		
ECMO-ECLS (*n* = 165)				*5.58 (2.29; 13.98)*	*5.1 (2.00; 13.41)*
Yes	25 (15%)	11 (9%)	14 (35%)		
No	140 (85%)	114 (91%)	26 (65%)		
Quantitative score of chest CT lesions (*n* = 167)	7.31 (6.62; 8.00)	6.58 (5.81; 7.33)	9.62 (8.09; 11.10)	*1.16 (1.06; 1.26)*	*1.91 (1.29; 2.91)*
ICU Therapy (*n* = 167)				*5.66 (2.57; 13.59)*	*5.29 (2.36; 12.83)*
Yes	79 (47%)	48 (38%)	31 (78%)		
No	88 (53%)	79 (62%)	9 (22%)		
ICU Duration (days *n* = 158)	8.30 (6.01; 10.95)	6.89 (4.44; 9.69)	13.25 (7.82; 19.83)	*1.02 (1.00; 1.04)*	*1.34 (1.04; 1.91)*
Mortality (*n* = 167)				*4.37 (2.00; 9.68)*	*4.22 (1.91; 9.42)*
Yes	38 (23%)	20 (16%)	18 (45%)		
No	129 (77%)	107 (84%)	22 (55%)		
Sepsis (*n* = 165)				*5.16 (2.19; 12.4)*	*4.75 (1.98; 11.5)*
Yes	28 (17%)	13 (10%)	15 (38%)		
No	137 (83%)	112 (90%)	25 (62%)		
C Reactive Protein (mg/l *n* = 144)	102 (88; 117)	88 (77; 102)	147 (108; 190)	*1.81 (1.25; 1.2.69)*	*1.96 (1.33; 3.00)*
Procalcitonin (mug/l *n* = 140)	0.78 (0.53; 1.06)	0.64 (0.37; 0.95)	1.23 (0.60; 1.98)	1.35 (0.95; 1.94)	1.31 (0.92; 1.92)
Pulmonary superinfection (*n* = 159)				*3.18 (1.47; 7.25)*	*3.10 (1.43; 7.11)*
Yes	78 (49%)	52 (43%)	26 (70%)		
No	81 (51%)	70 (57%)	11 (30%)		
D-Dimer (mg/l *n* = 138)	4.82 (3.54; 6.26)	4.53 (3.15; 6.11)	6.02 (3.51; 9.27)	1.18 (0.78; 1.69)	1.10 (0.71; 1.62)
PTT (sec *n* = 160)	36.61 (34.50; 39.18)	34.70 (32.70;37.04)	42.73 (36.50; 50.31)	*1.58 (1.11; 2.51)*	*1.55 (1.09; 2.41)*
INR (*n* = 160)	1.16 (1.09; 1.26)	1.09 (1.06; 1.13)	1.39 (1.13; 1.77)	*2.10 (1.10; 5.24)*	2.25 (1.21; 5.86)
Platelets (tsd/microl *n* = 167)	234.7 (218.6; 252.5)	221 (205; 238)	277 (242; 318)	*1.60 (1.14; 2.28)*	*1.46 (1.03; 2.10)*
Coagulopathy (*n* = 165)				2.40 (0.67; 8.01)	2.10 (0.58; 7.10)
Yes	12 (7%)	7 (6%)	5 (13%)		
No	153 (93%)	118 (94%)	35 (87%)		
Renal failure (*n* = 166)				*3.25 (1.57; 6.87)*	*3.27 (1.57; 6.98)*
Yes	60 (36%)	37 (29%)	23 (58%)		
No	106 (64%)	89 (71%)	17 (42%)		

Results highlighted in italics are statistically significant

*NIV* Non-invasive Ventilation,* ECMO* Extracorporeal Membrane Oxygenation,* ECLS* Extracorporeal Lung Support,* INR* International Normalized Ratio,* PTT* Partial Thromboplastin Time,* ICU* Intensive Care Unit

Acute cardiac events were not significantly associated with troponin T levels (OR: 1.003, 95% CI [0.996; 1.008]). ARDS was associated with more acute cardiac events (OR: 3.73, 95% CI [1.73; 8.30]) but not with troponin T levels (OR: 1.003, 95% CI [0.998; 1.010]). Mortality was associated with more acute cardiac events (OR: 4.23, 95% CI [1.92; 9.42]) and with higher levels of troponin T (OR: 1.007, 95% CI [1.001; 1.014]) (Table 7).

**Table 7:** Secondary outcome. Clinical characteristics of hospitalized COVID-19 patients

		Acute cardiac events		Without correction for vaccination status odds Ratio (± 95% CI)	With correction for vaccination status adj odds ratio (± 95% CI)
		No event	Event		
Troponin T (*n* = 99 ng/l)	40.32 (26.52; 56.72)	35.75 (22.36; 55.95)	56.33 (28.74; 85.51)	1.003 (0.997; 1.009)	1.003 (0.996; 1.008)

		ARDS		Without correction for vaccination status odds Ratio (± 95% CI)	With correction for vaccination status adj odds ratio (± 95% CI)
		No ARDS	ARDS		
Acute cardiac events (*n* = 167)				*4.05 (1.95; 8.71)*	*3.73 (1.73; 8.30)*
Yes	40 (24%)	15 (14%)	25 (40%)		
No	127 (76%)	90 (86%)	37 (60%)		
Troponin T (*n* = 99 ng/l)	40.32 (26.52; 56.72)	32.80 (21.54; 45.92)	56.83 (24.01; 105.24)	1.004 (0.998; 1.010)	1.003 (0.998; 1.010)

		Mortality		Without correction for vaccination status odds Ratio (±95% CI)	With correction for vaccination status adj odds ratio (±95% CI)
		Survived	Deceased		
Acute cardiac events (*n* = 167)				*4.38 (2.00; 9.68)*	*4.23 (1.92; 9.42)*
Yes	40 (24%)	22 (17%)	18 (47%)		
No	127 (76%)	107 (83%)	20 (53%)		
Troponin T (*n* = 99 ng/l)	40.32 (26.52; 56.72)	31.36 (20.52; 43.12)	80.68 (32.78; 148.61)	*1.006 (1.0007; 1.0131)*	*1.007 (1.001; 1.014)*

Results highlighted in italics are statistically significant

*ARDS* Acute Respiratory Distress Syndrome

## Discussion

In this study, COVID-19 vaccination was observed to lower the occurrence of acute cardiac events among hospitalized COVID-19 patients. Additionally, a significant reduction in levels of troponin T and LDH was noted in these patients.

Our analysis also revealed that vaccination substantially decreased the incidence of acute cardiac events in patients of middle age, whereas this benefit was not statistically significant in the elderly population. Conversely, a significant reduction in troponin T levels was seen in vaccinated older hospitalized COVID-19 patients, but such an effect was not statistically significant for middle-aged individuals.

Cardiac disease and type 2 diabetes were identified as significant risk factors for the development of acute cardiac events amid COVID-19 infection. In contrast, the presence of a rheumatologic disease appeared to have a protective effect against the onset of acute cardiac events.

Patients experiencing acute cardiac events, as opposed to those without, required more extensive medical interventions, including NIV, intubation with mechanical ventilation, and ECMO-ECLS. Additionally, this patient group exhibited a higher extent of chest CT lesions, necessitated longer and more intensive ICU care, showed a higher occurrence of sepsis, elevated CRP levels, more frequent pulmonary superinfections, and had increased mortality rates. Furthermore, laboratory findings indicated increased PTT levels, INR, and platelet counts, alongside a greater incidence of renal failure in COVID-19 patients with acute cardiac events.

Acute cardiac events were not significantly associated with troponin T levels. ARDS was associated with more acute cardiac events but not with troponin T levels. Mortality was associated with more acute cardiac events and with higher levels of troponin T.

COVID-19 has been associated with specific organ tropisms, yielding pronounced effects across various systems, particularly the cardiovascular system [5, 6]. In this study, 20–30% of patients experienced myocardial injury, as evidenced by increased troponin levels, presenting significant implications for long-term surveillance and outcomes among survivors. Moreover, the disease heightens the risk of acute cardiac events, such as myocardial infarction, heart failure, arrhythmias, and myocarditis [6].

Additionally, COVID-19 has demonstrated a notable direct viral affinity for cardiac tissue. Postmortem studies have revealed the presence of viral RNA in the cardiomyocytes of 35% of patients, underscoring this cardiotropic characteristic [5, 10]. The propensity for the virus to affect cardiac tissue is likely due to the high density of ACE2 receptors, which are particularly abundant in the heart and lungs [5].

Research has demonstrated that COVID-19 vaccination is effective in preventing infection from the virus [12, 13]. Furthermore, vaccination protects against a severe course of COVID-19, ARDS, mortality, and different organ system damage in hospitalized COVID-19 patients [20–23]. Based on this evidence, we hypothesized that COVID-19 vaccination would have a cardioprotective effect. This was measured directly by the onset of acute cardiac events (defined as myocardial infarction, heart failure, arrhythmia, and myocarditis) and indirectly through cardiac biomarkers, which are reliable indicators of cardiac injury [5, 24]. Our results support our hypothesis: vaccination resulted in a decrease in acute cardiac events and a reduction of troponin T and LDH levels among hospitalized COVID-19 patients. While troponin T is highly specific to myocardial injury, LDH is a nonspecific marker found in various cell types; thus, a decrease in troponin T levels in vaccinated patients signifies diminished cardiac injury, whereas a reduction in LDH levels indicates a broader decrease in systemic damage.

Aligned with findings from other studies that indicate a protective effect of COVID-19 vaccination, particularly in middle-aged patients against severe disease progression and ARDS [20, 21], we conducted a subgroup analysis comparing middle-aged and older patients. Unexpectedly, vaccination was found to reduce the onset of acute cardiac events mainly in middle-aged patients, whereas troponin T reduction was observed mainly in the older cohort. This is intriguing as, in other infections such as influenza, troponin levels are believed to correlate with acute cardiac events [24]. Our investigation into the relationship between acute cardiac events and troponin T levels did not yield a statistically significant result in our cohort. Initially counterintuitive, these results become consistent upon considering that the rise in troponin T levels might be primarily driven by a pathomechanism distinct from that of acute cardiac events. While middle-aged patients may experience severe disease outcomes and ARDS due to an exaggerated immune response, older individuals may not exhibit such an immunologic overreaction, possibly because of an age-related weaker immune system [20, 21].

Therefore, older patients might be more susceptible to direct viral damage due to the viral infection of various organs [20, 21].

Accordingly, an age-dependent increase in COVID-19 viral RNA within myocardial cells would be expected in postmortem analyses. Lindner et al. examined the association of cardiac viral infection in patients who died of COVID-19 [25]. Autopsy data from the study enabled us to calculate the mean age for patients with detectable and non-detectable viral RNA in cardiac tissue. The mean age for patients without cardiac COVID-19 RNA was 78.4 years (95% CI 73.0; 83.4), while it was 85.63 years (95% CI 83.17; 87.96) for those with detectable RNA. Consequently, patients with cardiac viral presence were on average 7.23 years older (95% CI 1.45; 13.33) than those without. This suggests that older patients may experience more direct viral damage to the heart, leading to elevated troponin T levels, which vaccination has the potential to mitigate. Moreover, the literature indicates that ACE2 expression, which is crucial for the virus to infect cells, tends to increase with age [26].

Conversely, acute cardiac events may stem from immune dysregulation and overreaction, commonly observed in patients with a severe manifestation of the disease, characterized by cytokine storms and ARDS [27]. These patients are typically of middle age [20]. Should this hypothesis hold, one would expect a correlation between ARDS and acute cardiac events rather than with troponin T levels. Our analyses confirmed that ARDS was significantly related to acute cardiac events but not to troponin T levels. This suggests that acute cardiac events are primarily a consequence of the systemic immune response's indirect effects on the cardiovascular system, risks of which appear to be mitigated by vaccination. Moreover, both acute cardiac events and higher troponin T levels seem to be associated with increased mortality.

In summary, patients in the middle-aged group are at a heightened risk for immunological overreactions, resulting in a more severe progression of the disease and a greater frequency of acute cardiac events. However, this risk appears to be diminished in those who have been vaccinated against COVID-19. Similarly, older individuals are more susceptible to direct viral damage to the heart, indicated by elevated troponin T levels, a risk that is also lessened in vaccinated patients.

Consistent with these findings, the onset of acute cardiac events is significantly associated with a more severe course of the disease (NIV, intubation and mechanical ventilation, ECMO-ECLS, ICU therapy, ICU duration), more inflammation (sepsis, higher CRP levels, pulmonary superinfection), organ failure (quantitative chest CT lesions, renal failure), and mortality.

To identify the predominant risk factors for acute cardiac events among hospitalized COVID-19 patients, we utilized a regularized logistic regression approach. This revealed two primary risk factors: type 2 diabetes and preexisting cardiac disease. Type 2 diabetes, in particular, stands out as a crucial risk factor for COVID-19, with numerous studies indicating its association with a more severe disease course, increased pulmonary and cardiac complications, and heightened mortality [21, 28, 29].

The increased vulnerability of patients with type 2 diabetes to COVID-19 may arise from several factors: there is an upregulation of ACE-2 and furin expression in these individuals, potentially easing the virus's entry into cells; additionally, their T-cell function is often compromised, possibly leading to further dysregulation of the immune system. Moreover, these patients frequently exhibit heightened levels of interleukin 6, which can exacerbate the cytokine storm seen in severe cases of COVID-19 [30]. Insulin resistance might also contribute to suboptimal immune responses, specifically during the acute phase of respiratory viral infections. Therefore, despite an increased baseline inflammatory state in individuals with insulin resistance, a paradoxically diminished immune response is often observed [31].

The association between preexisting cardiac disease and an increased risk of acute cardiac events in the context of COVID-19 is not unexpected, given the inherent vulnerabilities of these patients. This correlation has been consistently demonstrated across various studies [6].

The presence of rheumatologic diseases has been identified as a potential protective factor against the development of acute cardiac events. Prior research has explored the potential mitigating effects of agents used to treat rheumatic diseases on the onset and intensity of COVID-19. Findings suggest that treatments involving corticosteroids and TNF-α inhibitors may confer prophylactic benefits [32, 33]. Consequently, these agents might exert a cardioprotective effect in hospitalized COVID-19 patients, warranting additional research.

Associations were observed between acute cardiac events and elevated levels of INR, PTT, and platelet counts. Given the higher prevalence of cardiac disease—and subsequent use of coagulation-modifying medications—in patients who experienced acute cardiac events, we consider these elevated levels to be confounded by the underlying cardiac conditions and related treatments.

Finally, after highlighting two key aspects of COVID-19 research: the clinical impact of vaccination on acute cardiac events and cardiac biomarker levels, and the cellular mechanisms underlying disease pathogenesis and potential therapeutic targets, we want to point out that in parallel molecular investigations unveil a novel hub gene signature implicated in host immune responses and viral infection pathways. For example, Karami et al. used the bioinformatics tool weighted gene co-expression network analysis (WGCNA) combined with machine learning validation on bronchial epithelium cells and provided data on transcriptional profiling and treatment guidance [34]. Personalized treatment approaches may be achieved by combining information on vaccination status and gene expression.

## Limitations

This investigation was conducted as a retrospective, single-center cohort study. Due to its retrospective design, there exists an elevated possibility for confounding factors compared to prospective cohort studies. Nevertheless, rigorous measures have been implemented to identify and adjust for all conceivable confounding variables to ensure the veracity of our findings. Furthermore, retrospective studies are susceptible to biases and incomplete data capture, potentially leading to a biased estimation of outcomes. Conducting prospective cohort studies could mitigate biases associated with retrospective designs. However, no patients were lost between vaccination and follow-up, and we recorded no missing values for the primary outcome. In addition, we performed a post hoc power sensitivity analysis to control for type I and II errors when performing inference.

Moreover, it has to be considered that being conducted at a single tertiary referral hospital, the generalizability of study findings to a broader population is limited. Future studies should consider collaborating with multiple healthcare institutions across diverse geographical regions to increase the study population's diversity and improve the findings' external validity.

Within the healthcare system, specific confounding variables have arisen from policy decisions. Notably, in Germany, vaccination efforts were initially prioritized for the elderly population, highlighting the necessity of recognizing and adjusting for such confounding factors in our analysis. While the size of our cohort, comprising nearly 170 patients, was sufficient for examining most effects within our population, it presented limitations in thoroughly analyzing infrequent characteristics or events. For instance, the inclusion of only six pregnant women in our sample curtails the scope of our conclusions regarding the impact of vaccination on specific subgroups. Nonetheless, the beneficial effects of vaccination on the broader population were statistically significant, even after comprehensive adjustments for all confounding variables. However, due to the moderate sample size, we were not able to differentiate between various COVID-19 vaccination types, potentially overlooking differences in efficacy and adverse event profiles. Future studies should stratify analyses based on vaccination types to elucidate potential variations in outcomes. Additionally, exploring the impact of vaccination dosing schedules and booster shots could provide valuable insights into long-term vaccine effectiveness. The study focused primarily on acute cardiac events and troponin levels, potentially overlooking other clinically relevant outcomes such as long-term cardiovascular sequelae or mental health effects. Incorporating a broader range of outcomes in future studies, including mental health assessments, long-term cardiovascular monitoring, and quality of life measures, would provide a more comprehensive understanding of the impact of COVID-19 and vaccination.

The evolving mutations of the virus add complexity to the task of generalizing our findings to new variants and strains that may emerge in the coming years. Nonetheless, by conducting iterative analyses across various independent studies and at different intervals, we might be able to infer broader principles that could inform future meta-analyses.

## Conclusion

COVID-19 vaccination exhibits a cardioprotective effect in hospitalized COVID-19 patients. This effect was quantified directly by a decrease in acute cardiac events and indirectly by lowered troponin T levels among vaccinated COVID-19 patients.

The observed decrease in acute cardiac events was notably more substantial in middle-aged patients, suggesting that these events may stem from systemic dysregulation and immunologic overreactions, which are more prevalent in middle-aged individuals with COVID-19. In contrast, the significant reduction in troponin T levels was primarily seen in older patients, potentially indicative of direct viral infection of the cardiomyocytes, a condition that appears to be more common in the elderly COVID-19 population. Moreover, both troponin T elevations and acute cardiac events have been associated with increased mortality rates.

Therefore, our data suggest that middle-aged and older patients both profit from COVID-19 vaccination, probably due to different mechanisms of cardiac system damage.

## Supplementary Information

Below is the link to the electronic supplementary material.

Supplementary Figure 1. Percentage of acute cardiac events (ACE) in non-vaccinated and vaccinated patients with COVID-19.
Supplementary file1 (EPS 1997 KB)

Supplementary Table 1. Acute cardiac events (ACE) in vaccinated and non-vaccinated patients with COVID-19.
Supplementary file2 (DOCX 30 KB)

Supplementary Table 2. Influence of coronary calcification on acute cardiac events and Troponin T levels.
Supplementary file3 (DOCX 25 KB)

## Data Availability

The datasets used and analyzed during the current study are available from the corresponding author on reasonable request.

## Abbreviations

ACE2: Angiotensin converting enzyme 2
ARDS: Acute respiratory distress syndrome
CI: Confidence interval
CK: Creatine kinase
COVID-19: Coronavirus disease
CT: Computed tomography
ECLS: Extracorporeal lung support
ECMO: Extracorporeal membrane oxygenation
ICU: Intensive care unit
INR: International normalized ratio
LASSO: Least absolute shrinkage and selection operator
LDH: Lactate dehydrogenase
nt-pro-BNP: N-terminal pro b-type natriuretic peptide
OR: Odds ratio
PTT: Partial thromboplastin time
RNA: Ribonucleic acid
RR: Relative risk
RT-PCR: Reverse transcriptase polymerase chain reaction
