# Supplementary material for: Cardioprotective effects of vaccination in hospitalized patients with COVID-19
Source: Clin Exp Med. 2024 May 17;24(1):103. doi: 10.1007/s10238-024-01367-3 (PMC11101587; doi:10.1007/s10238-024-01367-3)
Supplement: Supplementary file 1 [file 10238_2024_1367_MOESM2_ESM.docx]

no

ACE (without Oncological Disease n=140)

yes

no
